# Discriminating Small-Sized (2 cm or Less), Noncalcified, Solitary Pulmonary Tuberculoma and Solid Lung Adenocarcinoma in Tuberculosis-Endemic Areas

**DOI:** 10.3390/diagnostics11060930

**Published:** 2021-05-21

**Authors:** Jingping Zhang, Tingting Han, Jialiang Ren, Chenwang Jin, Ming Zhang, Youmin Guo

**Affiliations:** 1 Department of Radiology, The First Affiliated Hospital of Xi’an Jiaotong University, 277 West Yanta Road, Xi’an 710061, China; zhangjp@xjtufh.edu.cn (J.Z.); croissanthtt@stu.xjtu.edu.cn (T.H.); zhangming01@xjtu.edu.cn (M.Z.); cjr.guoyoumin@vip.163.com (Y.G.); 2 GE Healthcare China, Daxing District, Tongji South Road No.1, Beijing 100176, China; jialiang.ren@ge.com

**Keywords:** noncalcified, solitary tuberculoma, solid adenocarcinoma, solitary pulmonary nodule, computed tomography morphological features

## Abstract

Background. Pulmonary tuberculoma can mimic lung malignancy and thereby pose a diagnostic dilemma to clinicians. The purpose of this study was to establish an accurate, convenient, and clinically practical model for distinguishing small-sized, noncalcified, solitary pulmonary tuberculoma from solid lung adenocarcinoma. Methods. Thirty-one patients with noncalcified, solitary tuberculoma and 30 patients with solid adenocarcinoma were enrolled. Clinical characteristics and CT morphological features of lesions were compared between the two groups. Multivariate logistic regression analyses were applied to identify independent predictors of pulmonary tuberculoma and lung adenocarcinoma. Receiver operating characteristic (ROC) analysis was performed to investigate the discriminating efficacy. Results. The mean age of patients with tuberculoma and adenocarcinoma was 46.8 ± 12.3 years (range, 28–64) and 61.1 ± 9.9 years (range, 41–77), respectively. No significant differences were observed concerning smoking history and smoking index, underlying disease, or tumor markers between the two groups. Univariate and multivariate analyses showed age and lobulation combined with pleural indentation demonstrated excellent discrimination. The sensitivity, specificity, accuracy, and the area under the ROC curve were 87.1%, 93.3%, 90.2%, and 0.956 (95% confidence interval (CI), 0.901–1.000), respectively. Conclusion. The combination of clinical characteristics and CT morphological features can be used to distinguish noncalcified, solitary tuberculoma from solid adenocarcinoma with high diagnostic performance and has a clinical application value.

## 1. Introduction

Tuberculosis (TB) remains the top infectious killer worldwide, and China has the second-highest TB burden worldwide [1]. Tuberculoma is seen in approximately 9% of tuberculosis patients [2]. Typically, pulmonary tuberculomas are round, well-defined lesions with small satellite lesions in the immediate vicinity of the main lesion, and calcification can be seen in 20% to 30% of them [3]. However, they can sometimes present as noncalcified, solid solitary pulmonary nodules (SPNs) with atypical imaging characteristics such as lobulation, spiculation, vessel convergence, and pleural indentation, signs that are consistent with a lung malignancy, thus representing a diagnostic dilemma for clinicians. Previous studies have reported that pulmonary tuberculosis resulted in about 57.1% to 92.0% false-positive diagnoses of primary lung cancer in pulmonary TB endemic regions, and pulmonary tuberculoma was the most common type of benign SPN [4]. In contrast, lung adenocarcinomas (LACs) are the most common pathological type of malignant SPNs [4,5,6]. Accurate differentiation between pulmonary tuberculoma and lung adenocarcinoma is pivotal because this prompts clinicians to develop an appropriate management plan. For pulmonary tuberculoma, this involves the avoidance of unnecessary therapeutic procedures, while for lung adenocarcinoma, this improves the treatment outcome and prognosis.

Although there have been several studies concerning the differential diagnosis of benign and malignant SPNs, few studies have focused on discriminating small-sized (2 cm or less), noncalcified, solitary pulmonary tuberculoma and solid lung adenocarcinoma. Most of the previous studies concerning distinguishing pulmonary tuberculoma from lung cancer are related to 18F-fluorodeoxyglucose (18F-FDG) positron emission tomography/computed tomography (PET/CT), which is, to some extent, nonspecific and unfortunately very costly [4,7,8]. Contrast-enhanced dynamic CT has also been used to discriminate tuberculoma from lung malignancy in few studies. Most tuberculoma cases often show no enhancement and a flat time-density curve, which is very different from lung malignancy [9]. However, there are also many limitations with this method, such as the relatively low temporal and spatial resolution, the selection of the region of interest (ROI), and the size of the lesion. Moreover, pulmonary tuberculoma demonstrates various enhancements depending on the inflammatory phases of this disease. Patients with active tuberculomas usually have high peak height values of the time-density curve, much like lung malignancies [10]. CT remains a primary first-line imaging modality for pulmonary disease and is recommended for lung cancer screening; it can provide essential diagnostic and differential information. Though low-dose CT is currently recommended to reduce radiation exposure, it seems to be less sensitive and accurate than is standard-dose CT in some instances. Previous studies suggested that some variation occurs in interpreting low-dose CT scans among radiologists [11]. Besides, incidental, indeterminate SPNs are usually encountered in lung screening or medical examinations in daily clinical practice. Thus, the purpose of this study was to evaluate the detailed clinical and imaging features to screen for the critical characteristics for discriminating small-sized pulmonary tuberculoma and solid lung adenocarcinoma and build an accurate, convenient, and clinically practical diagnostic model.

## 2. Materials and Methods

### 2.1. Subjects

**Figure 1:**
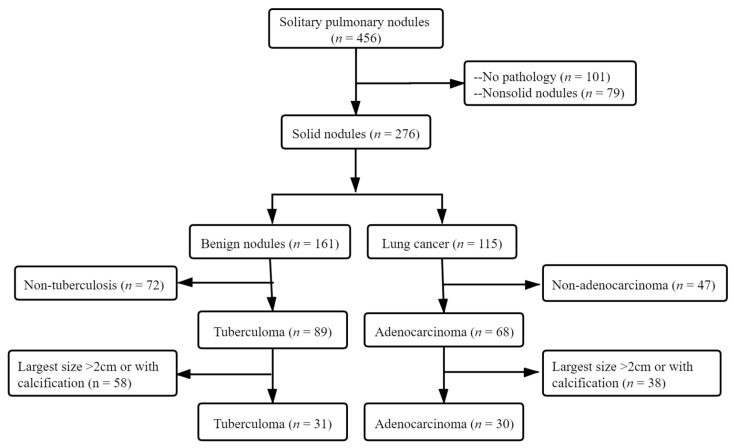
The flowchart for selecting the study population.

From January 2014 to December 2019, we retrospectively enrolled a total of 456 consecutive patients with indeterminate SPNs. Among them, 101 patients were initially excluded due to no histopathological examinations, and 79 patients were excluded for nonsolid nodules. Of the 276 patients who were presumptively diagnosed with lung malignancy and had a definite pathological diagnosis depending on percutaneous transthoracic needle biopsy or pulmonary lobectomy excision, 161 patients were proven to have benign lesions, and 115 patients were confirmed to have lung cancer. Of the 161 patients with benign nodules, 72 patients with non-tuberculoma and 58 patients with tuberculoma larger than 2 cm or with calcification were excluded; of the 115 patients with lung malignancy, 47 patients with non-adenocarcinoma and 38 patients with adenocarcinoma larger than 2 cm or with calcification were excluded. In total, 31 patients with noncalcified, solitary tuberculoma and 30 patients with solid adenocarcinoma were included in this study. The patient selection pipeline is depicted in a flow diagram (Figure 1).

The patients’ electronic medical records were reviewed for clinical data, including patients’ sex, age, smoking history, smoking quantity, medical history (emphysema, diabetes, previous malignancy history), and the serum level of tumor markers associated with adenocarcinoma (carbohydrate antigen 125 (CA125) and carcinoembryonic antigen (CEA)). Clinical data collection was conducted by two physicians.

### 2.2. CT Imaging

Chest CT scans were conducted with a 64-multidetector CT scanner (Brilliance 64; Philips, Eindhoven, The Netherlands) with the following protocol parameters: patients were in a supine position, the range was from the apex to the base of the lung, including the chest wall and axillary fossa; additional parameters were as follows: 120 KVp; 250 mAs; collimation, 0.625 mm; slice thickness and interval for axial images, 3 mm/3 mm.

The CT morphological features were evaluated in the lung window (level, −500 HU; width, 1500 HU). The interpretation of CT images was conducted by two experienced chest radiologists who were blind to the pathologic results of the lesions, and all disagreements, if any, were resolved through consensus. The CT morphological features, including the maximum transverse size of the nodule (measured on lung window), shape (round like, irregular), margin (clear, hazy), spiculation (present, absent), lobulation (present, absent), pleural indentation (present, absent), air bronchogram (present, absent), cavity or vacuole (present, absent), blood vessel convergence (present, absent), perilesional GGO (present, absent), perilesional tree-in-bud pattern (present, absent), and the location in the lung, were evaluated with reference to the Fleischner Society’s glossary of terms for thoracic imaging [12]. Especially for the nodule size measurement, we choose the maximum transverse diameter instead of nodule volume because current nodule management is still based on nodule diameter, and volumetric measurements are dependent on specific software that is not very convenient [13]. All CT images were reviewed in random order.

### 2.3. Statistical Analysis

All statistical analyses were performed with R software (v.4.0.5.; R Core Team, R: A Language and Environment for Statistical Computing 2013, Available at http://www.r-project.org/, accessed on 31 March 2021). Categorical variables were expressed as frequencies and percentages, and nonnormally distributed continuous variables were expressed as the median [Q1, Q3]. Statistical tests were conducted for between-group differences in the CT morphological features, clinical characteristics, and tumor markers by using the Pearson’s chi-square test and Mann–Whitney rank-sum test as appropriate. The results were considered statistically significant at a *p*-value of less than 0.05, and all reported *p*-values are two-tailed. The variables with a *p*-value less than 0.05 in the univariate analysis were included in a stepwise multivariate logistic regression analysis to choose the optimal predictors for tuberculoma. ROC curve analysis was conducted for the variables that exhibited statistically significant differences in the multivariate analysis and was used to derive the sensitivity, specificity, and accuracy of the selected variables in predicting pulmonary tuberculoma and lung adenocarcinoma.

## 3. Results

### 3.1. Clinical Characteristics and Tumor Markers

Thirty-one patients with noncalcified pulmonary tuberculoma and thirty patients with solid lung adenocarcinoma were enrolled. The patients’ clinical characteristics are shown in Table 1. Of the 31 patients with pulmonary tuberculoma, the mean age was 46.8 ± 12.3 years (range, 28–64 years); 25 (80.6%) patients were < 60 years, and there were slightly more males (18/31, 58.1%) than females (13/31, 41.9%). There was no difference in sex distribution in patients with lung adenocarcinoma, and the mean age was 61.1 ± 9.9 years (range, 41–77 years); only 11 (36.7%) patients were < 60 years. There was an evident statistically significant difference between patients with tuberculoma and adenocarcinoma regarding age (*p*-value = 0.001); the patients with lung adenocarcinoma were much older than those with tuberculoma. The numbers of smokers among pulmonary tuberculoma and lung adenocarcinoma patients were 11 (35.5%) and 12 (40.0%), with mean smoking indexes of 166.1 ± 285.6 and 349.7 ± 789.6, respectively, but there were no significant differences between the two groups. Furthermore, no significant differences were observed concerning diabetes and emphysema history between patients with tuberculoma and adenocarcinoma (*p*-value = 0.955, 0.215, respectively). In reference to tumor markers, none of these showed statistically significant differences between the two groups; all the *p*-values were more than 0.05 (Table 1); some patients had no detected tumor markers before they underwent surgery or percutaneous transthoracic needle aspiration biopsy.

### 3.2. CT Morphological Features

The CT morphological features of tuberculoma and adenocarcinoma are summarized in Table 2. The maximum diameter of tuberculomas ranged between 0.91 cm and 2.00 cm, and the mean value was 1.51 cm, while the maximum diameter of the adenocarcinomas ranged between 0.64 cm and 2.00 cm, and the mean value was 1.53 cm. Univariate analysis of the CT morphological features showed that there were no significant differences between patients with tuberculoma and adenocarcinoma in terms of nodule shape, location, spiculation, cavity, vacuole, air bronchogram, perilesional GGO, and lymphadenopathy (*p*-value > 0.05, Table 2), yet both patients with tuberculoma and patients with adenocarcinoma showed obvious upper lobe distribution preponderance (64.5%, and 46.7%, respectively). Other features, such as margin, lobulation, pleural indentation, perilesional tree-in-bud pattern, blood vessel convergence, and satellite lesions demonstrated obvious statistically significant differences (*p*-value < 0.05). Patients with tuberculoma had a significantly higher frequency of hazy margins (12.9% vs. 0.0%, *p*-value = 0.042), satellite lesions (29.0% vs. 3.3%, *p*-value = 0.007), and perilesional tree-in-bud patterns (25.8% vs. 0.0%, *p*-value < 0.001) and a relatively lower frequency of lobulation (29.0% vs. 93.3%, *p*-value < 0.001), pleural indentation (32.3% vs. 83.3%, *p*-value < 0.001), and blood vessel convergence (71.0% vs. 100.0%, *p*-value = 0.001) than did patients with adenocarcinoma.

**Figure 2:**
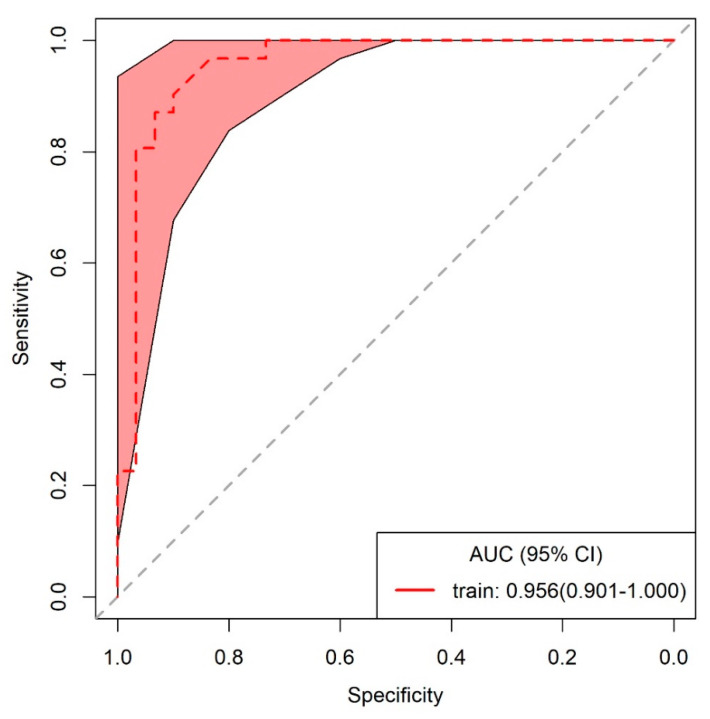
Receiver operating characteristic (ROC) curve applied to distinguish tuberculoma from lung adenocarcinoma. AUC: area under the ROC curve; 95% CI: confidence interval.

**Figure 3:**
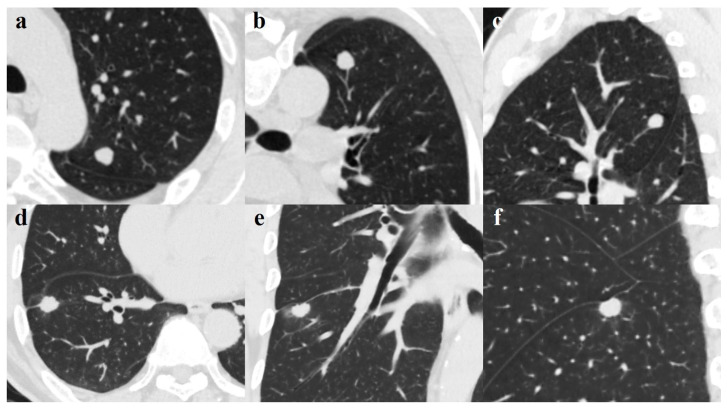
(**a**–**c**). A 49-year-old man with pulmonary tuberculoma in the upper lobe of the left lung. Axial image (**a**) showing a round-like well-defined solid nodule measuring 1.14 cm; blood vessel convergency can be seen on the reconstructed coronal (**b**) and sagittal (**c**) views. The lesion was confirmed on pathological diagnosis as a tuberculoma. d-f. A 65-year-old man with lung adenocarcinoma in the right lower lobe. Axial image (**d**) demonstrates a well-defined juxta-fissural solid nodule measuring 1.47 cm with lobulated margin, short burr, and pleural indentation sign. Perilesional ground-glass opacification and a pleural indentation sign can be seen on the coronal (**e**) and sagittal (**f**) views. The lesion was proven on pathological diagnosis to be a moderately differentiated adenocarcinoma.

Stepwise multivariate logistic regression analysis with tuberculoma as an outcome was performed to derive the optimal variables that could discriminate tuberculoma from adenocarcinoma and to build the model. Variables with significant differences in the univariate analysis were all included. Ultimately, age, lobulation, and pleural indentation remained significant and were selected for model building, and an ROC curve with a statistically significant area under the curve (AUC) was obtained (0.956; 95% CI 0.901–1.000) (Figure 2). The sensitivity, specificity, and accuracy in discriminating tuberculoma and adenocarcinoma were 87.1%, 93.3%, and 90.2%, respectively; the positive predictive value (PPV) was 93.1% (95% CI 79.8–93.9), and the negative predictive value (NPV) was 87.5% (95% CI 85.7–88.2). These analyses indicate that age in combination with lobulation and pleural indentation showed an excellent capacity in discriminating noncalcified, solitary pulmonary tuberculoma and solid adenocarcinoma with a maximum diameter of 2 cm or less. The Hosmer–Lemeshow test (*p*-value > 0.05) and assessment of bootstrap calibration curves suggested an adequate model fit. Figure 3 provides representative chest computed tomography images of noncalcified pulmonary tuberculoma and solid adenocarcinoma.

## 4. Discussion

In tuberculosis-endemic areas, the diagnosis of pulmonary tuberculoma can cause a great deal of trouble to clinicians when encountering an indeterminate SPN since pulmonary tuberculoma shares some presupposed malignant morphological features with lung cancer. Currently, radiomics and artificial intelligence are promising tools for differentiating between benign and malignant nodules on CT images and have achieved some promising results [14,15]. However, while intuitively appealing, these approaches have not been widely promoted in clinical practice because of their complex practical application and demanding technical requirements. In this retrospective study, we assessed the value of clinical characteristics and tumor markers combined with CT morphological features in distinguishing noncalcified, solitary pulmonary tuberculoma from solid adenocarcinoma.

Concerning the clinical characteristics, patients with tuberculoma tended to be much younger (the mean age was 46.8 ± 12.3 years) than those with adenocarcinoma (the mean age was 61.1 ± 9.9 years), which is consistent with previous studies [16,17,18]. Other characteristics, such as sex distribution, smoking history, and underlying disease, all showed no obvious differences between the two groups in our study. It is not surprising that smoking history showed no difference between the two groups since smoking is a common risk factor shared by both diseases. Previous studies reported that people with a history of diabetes are more vulnerable to tuberculosis because of impairment of the immune system [17,19,20]; however, only a small number of patients with tuberculoma were complicated with diabetes in our study, which may be due to our small sample size and inevitable selection bias.

Serum levels of tumor markers are commonly measured in clinical practice for auxiliary diagnosis of lung cancer. Generally, lung cancers are accompanied by high levels of tumor markers; however, it has been reported that in some benign diseases, such as tuberculosis, abnormal concentrations of these tumor markers can also be detected, thus leading to false-positive diagnoses [21,22]. In our cohort, both CEA and CA125 levels were normal in patients with tuberculoma and adenocarcinoma, and there was no significant difference between the two groups, which may be because of the small sample size and early stage of the malignant lesions. Moreover, many patients had no detected tumor markers before they underwent operation or percutaneous transthoracic needle aspiration biopsy, indicating that the tumor markers are, to some extent, not sensitive and specific in distinguishing benign nodules from malignant nodules.

CT morphological features are vital for radiologists in distinguishing benign and malignant solid nodules in daily clinical practice and have been used as prognostic factors for patients with lung cancer [23]. Unfortunately, there are considerable overlaps between tuberculoma and lung cancer regarding CT morphological features, such as spiculation, lobulation, pleural indentation, or vessel convergence. In our study, univariate analysis showed that pulmonary tuberculoma tended to be less lobulated, with less pleural indentation and vessel convergence, yet more satellite lesions and perilesional tree-in-bud patterns than seen in lung adenocarcinoma. However, it is noteworthy that the frequency of vessel convergence was significantly higher in patients with tuberculoma (71.0%), indicating that this sign is very nonspecific. Still, though satellite lesions are generally considered as characteristic of benign nodules, their frequency in solitary tuberculoma was relatively low in our study (29.0%); moreover, satellite lesions can also be identified in about 10% of lung adenocarcinoma [24]. The tree-in-bud pattern was initially described in the case of endobronchial spread of pulmonary tuberculosis [25]. Although this sign has subsequently been recognized in a wide variety of entities and is nonspecific for tuberculosis, it is rarely seen in malignancies except neoplastic pulmonary emboli [26]. In our opinion, when encountering an indeterminate solid SPN with the perilesional tree-in-bud pattern, a benign nodule, especially tuberculoma, should be considered first in tuberculosis-endemic areas. There is a slight flaw with using this sign: As its frequency was relatively low in solitary tuberculoma in our study (25.8%) its application in daily clinical practice is limited; this may because the tree-in-bud pattern is in general a characteristic of active disease, whereas tuberculomas are most often the result of healed primary PTB or a result of reactivation [3,27]. Further multivariate analysis showed that lobulation and pleural indentation are useful CT morphological features for differentiating tuberculoma and adenocarcinoma.

Lobulation is a well-known sign associated with malignancy. The lobulated contour of a malignant nodule is usually caused by a heterogeneous growth rate, while in benign nodules, it is a result of hyperplasia of adjacent connective tissue and cicatricial contraction [28]. More than 90% of adenocarcinomas in our cohort exhibited changes in lobulation, whereas the occurrence rate of lobulation in tuberculoma was only 29%. This result is in concordance with previous studies [23,29]. In a study derived from the NELSON trial, Xu and his colleagues found that in solid noncalcified nodules larger than 50 mm^3^, size rather than shape, margin, or nodule density is the main predictive factor of malignancy; though to a lesser extent than size, lobulation can also increase the likelihood that a nodule is malignant [30]. Current studies have reported that lobulation is associated with the histological subtypes of early-stage lung adenocarcinoma and can serve as a predictive factor for prognosis [31].

Pleural indentation is a key radiological sign that suggests the possibility of malignant and visceral pleural invasion. The mechanism of pleural indentation is a combination of contractile changes within the tumor and compensatory expansion of peritumoral lung parenchyma to fill the space between the areas of retracted visceral pleura [32]. However, in some cases, inflammation, and fibrosis, such as that in tuberculosis, could also affect the pleura [33]. This sign should be evaluated with care because the relationship between the SPN and the pleura can be divided into several different conditions, such as pleural attachment, pleural indentation, or both, and different relationships may correlate with different incidences of visceral pleural invasion [34,35]. In our study, both pleural indentation alone and concurrent pleural indentation and attachment were identified as pleural indentation. Pleural indentation was identified in 32.3% of patients with tuberculoma versus 83.3% of patients with adenocarcinoma (*p* < 0.001). This result is in line with those of some previous studies. Harders et al. reported that pleural indentation could be recognized in 58% of malignant SPNs and 31% of benign SPNs and could serve as a highly significant predictor of malignancy with a positive likelihood ratio of two [36]. Lang et al. found that pleural indentation could be seen in 36.4% of patients with pulmonary tuberculoma, which is similar to our result; however, a relatively low frequency (37%) was observed in patients with lung cancer [17]. This may be due to the different inclusion criteria since in our study, only peripheral lung adenocarcinoma was included, but in theirs, they did not make a distinction between the different pathological types of lung cancer or peripheral and central lung cancer.

## 5. Limitation and Future Work

The present study has several limitations. First, the sample size of our study was small due to the strict inclusion criteria, and the number of variables evaluated was relatively high. Second, selection bias cannot be ignored since only patients who had pathologic results after percutaneous transthoracic needle aspiration biopsy or surgery were included; moreover, only lung adenocarcinoma and tuberculoma without calcification were observed, so the results should be interpreted carefully. Third, our study was conducted retrospectively in a single institution; thus, a definitive conclusion could not be reached. Further work in a prospective, multicenter design with a large cohort is needed to validate and expand upon the results.

## 6. Conclusions

In conclusion, our data demonstrated that age and lobulation combined with pleural indentation have a high sensitivity and specificity in distinguishing noncalcified, solitary pulmonary tuberculoma from solid adenocarcinoma with a maximum diameter of 2 cm or less, and all the characteristics mentioned above are very convenient to apply in routine clinical practice. Features that suggest adenocarcinoma include older age, lobulated contour, and pleural indentation; otherwise, benign nodules, especially tuberculoma, should be considered in tuberculosis-endemic areas. If satellite lesions or perilesional tree-in-bud pattern are observed, the diagnosis is more likely to be tuberculoma. However, it is essential to note that this conclusion must be interpreted with caution since, in an area where tuberculosis is less widespread, such as in western countries, this may not be applicable. In addition, the final diagnosis relies on histopathological verification.

## Figures and Tables

**Table 1:** Clinical characteristics and tumor markers in patients with tuberculoma vs. those with adenocarcinoma.

Characteristics	Tuberculoma no. (%)	Adenocarcinoma no. (%)	Total no. (%)	*p*-Value
**No.**	31	30	61	
**Sex**				0.527 ^1^
Female	13 (41.9%)	15 (50.0%)	28 (45.9%)	
Male	18 (58.1%)	15 (50.0%)	33 (54.1%)	
**Age**				<0.001 ^2^
Mean (SD)	46.8 (12.3)	61.1 (9.9)	53.8 (13.2)	
Median [Q1, Q3]	50.0 [35.0, 57.5]	61.5 [53.5, 70.0]	56.0 [44.0, 63.0]	
Range	28.0–64.0	41.0–77.0	28.0–77.0	
**Smoker**	11 (35.5%)	12 (40.0%)	23 (37.7%)	0.716 ^1^
**Smoking index**				0.545 ^2^
Mean (SD)	166.13 (285.59)	349.67 (789.56)	256.39 (592.16)	
Median [Q1, Q3]	0.00 [0.00, 175.00]	0.00 [0.00, 425.00]	0.00 [0.00, 200.00]	
Range	0.00–900.00	0.00–4000.00	0.00–4000.00	
**Underlying disease**				
Emphysema	6 (19.4%)	10 (33.3%)	16 (26.2%)	0.215 ^1^
Diabetes	5 (16.1%)	5 (16.7%)	10 (16.4%)	0.955 ^1^
**Tumor marker**				
CA125, >35 U/mL				0.063 ^2^
N-Miss	21	13	34	
Median [Q1, Q3]	7.80 [4.73, 11.70]	12.10 [10.10, 17.20]	10.70 [7.80, 16.20]	
CEA, >5 ng/mL				0.057 ^2^
N-Miss	16	8	24	
Median [Q1, Q3]	1.63 [1.25, 2.26]	2.21 [1.72, 4.72]	1.97 [1.57, 3.65]	

^1^ Pearson’s chi-square test; ^2^ Mann–Whitney rank sum test. Abbreviations: no., number; SD, standard deviation; N-Miss, number of missed; CEA, carcinoembryonic antigen.

**Table 2:** CT morphological features in patients with tuberculoma vs. those with adenocarcinoma.

CT Features	Tuberculoma no. (%)	Adenocarcinoma no. (%)	Total no. (%)	*p*-Value
**No.**	31	30	61	
**Maximum diameter**				0.756 ^2^
Median [Q1, Q3]	1.45 [1.23, 1.86]	1.57 [1.38, 1.83]	1.520 [1.29, 1.8]	
**Irregular shape**	17 (54.8%)	21 (70.0%)	38 (62.3%)	0.222 ^1^
**Clear margin**	27 (87.1%)	30 (100.0%)	57 (93.4%)	0.042 ^1^
**Lobulation**	9 (29.0%)	28 (93.3%)	37 (60.7%)	<0.001 ^1^
**Spiculation**	26 (83.9%)	29 (96.7%)	55 (90.2%)	0.093 ^1^
**Perilesional GGO**	7 (22.6%)	7 (23.3%)	14 (23.0%)	0.944 ^1^
**Pleural indentation**	10 (32.3%)	25 (83.3%)	35 (57.4%)	<0.001 ^1^
**Satellite lesions**	9 (29.0%)	1 (3.3%)	10 (16.4%)	0.007 ^1^
**Cavity**	3 (9.7%)	3 (10.0%)	6 (9.8%)	0.966 ^1^
**Vacuole**	4 (12.9%)	1 (3.3%)	5 (8.2%)	0.173 ^1^
**Air bronchogram**	3 (9.7%)	2 (6.7%)	5 (8.2%)	0.668 ^1^
**Vessel convergence**	22 (71.0%)	30 (100.0%)	52 (85.2%)	0.001 ^1^
**Perilesional tree-in-bud pattern**	8 (25.8%)	0 (0.0%)	8 (13.1%)	<0.001 ^1^
**Lymphadenopathy**	1 (3.2%)	2 (6.7%)	3 (4.9%)	0.534 ^1^
**Location**				0.389 ^1^
LUL	7 (22.6%)	6 (20.0%)	13 (21.3%)	
LLL	7 (22.6%)	6 (20.0%)	13 (21.3%)	
RUL	13 (41.9%)	8 (26.7%)	21 (34.4%)	
RLL	3 (9.7%)	6 (20.0%)	9 (14.8%)	
RML	1 (3.2%)	4 (13.3%)	5 (8.2%)	

^1^ Pearson’s chi-square test; ^2^ Mann–Whitney rank sum test. Abbreviations: no., number; SD, standard deviation; RUL, right upper lobe; RML, right middle lobe; RLL, right lower lobe; LUL, left upper lobe; LLL, left lower lobe; GGO, ground-glass opacity.

## Data Availability

The data presented in this study are available on request from the corresponding author. The data are not publicly available due to ethical concern.
